# 89. Alkaptonuria: a case report

**DOI:** 10.1093/rap/rky034.052

**Published:** 2018-09-20

**Authors:** Siwalik Banerjee, Himashi Anver, Jonathon Pinnell, Phillip Perkins

**Affiliations:** Rheumatology, University Hospital of Coventry and Warwickshire, Coventry, UNITED KINGDOM

**Introduction:** Alkaptonuria is a rare genetic disorder of autosomal recessive inheritance, with a worldwide prevalence of 1 in 250,000 to 1000000. It is caused by a mutation in the phenylalanine and tyrosine catabolism pathway, characterised by accumulation of Homogentisic acid (HGA) in the connective tissues, and increased excretion of HGA in urine. HGA undergoes oxidation to form a brown black pigment, which results in the characteristic cola coloured urine. HGA also polymerises in the body to form a brown black pigment which is deposited mostly in the connective tissues, causing widespread joint and cartilage damage - a process known as ochronosis. Here we present a case of advanced ochronosis in a 52 year Caucasian gentleman.

**Case description:** SL was born in 1966 to English parents. A few weeks after his birth his mother noticed dark purple stains in his nappies. He was initially investigated for possible porphyria. As the urine porphobilinogen test was negative, he was tested for urine HGA levels, and was diagnosed with alkaptonuria. However, the diagnosis was not explained to the family clearly, and he was never followed up. SL was asymptomatic up to the age of 22 . He was extremely athletic and played football and rugby, and worked as an engineer. In 1988, he was admitted to the hospital with duodenal ulcer. At that time he was investigated again for dark coloured urine and the diagnosis of alkaptonuria was reaffirmed. It was explained to him that he may develop mild arthritis in future. Since 1988, he started to have low back pain, and complained of progressive low- back stiffness. The back pain increased with rest and improved with exercise, mimicking an inflammatory low back pain. In 1997 he collapsed at work with severe back pain and was hospitalised. The treating physicians suspected Ankylosing spondylitis, however his MRI scan did not show any sacroilits, instead showed intervertebral disc calcification in the lumbar region. Due to Progressively increasing back pain, he lost his job. Since then he has been under regular rheumatology follow up. Around this time, his sweat turned brown, he had dark cerumen and developed a dark coloured stiff pinna. A year later he developed strangury and dysuria, and was investigated for urinary tract infection. His cystoscopy revealed a dark bladder mucosa. He was diagnosed with prostatic stones, and continued passing small gravel-like stones. He was started on alfuzosin. Due to his ongoing back pain, he was under the pain team and received regular facet joint injections. At the age of 32 he developed bilateral knee pain and was diagnosed with meniscal damage. Arthroscopy revealed dark coloured cartilage and dark intra articular debris. To this date he has had four arthroscopic interventions in each knee. Arthroscopy only gave him temporary symptom relief. In 2001 he volunteered for a trial in the National Institute of Health, United States and was extensively investigated. The trial began in 2005 and he was started on nitisinone, a drug licensed for the treatment of hereditary tyrosinemia type 1. He travelled to the United States four times a year for the trial. He felt remarkably well when on nitisinone, and his back, hip and knee pain reduced by 60 percent, his urine and sweat colour changed to normal, and the brown pigmentation and stiffness of pinna disappeared. He stopped passing stones in urine. His urinary HGA reduced by 95%. However, the trial ended in 2007 and the results were inconclusive. He was off nitisinone, and he was back to his previous state. In 2012 he was reviewed in the Royal Liverpool University Hospital, and since then he is under treatment in the National AKU Centre ( established in 2013). He is at present on nitisinone 2mg/day, and reports significant symptom improvement. He remains under regular rheumatology review in the University Hospital Coventry and Warwickshire. In 2016 he was diagnosed with osteoporosis and started on zolindronic acid. He also has obstructive sleep apnoea and uses a CPAP machine. He has developed fusion of cervical vertebrae and has limited neck rotation. He has cervical and lumbar radiculopathy and receives yearly nerve blocks. He uses lignocaine patches, apart from regular analgesia. He is on a low protein diet (.5gm/Kg). Mr SL remains independent with activities of daily living. He has two daughters, both of whom are carriers. His parents also underwent genetic testing, and were diagnosed to be carriers, as expected in a true autosomal recessive condition. His sister is symptom free, and not keen on genetic testing. Now 52, Mr SL keeps active and helps to raise awareness of alkaptoniria among general people.

**Discussion:** Alkaptonuria can cause severe destructive spondyloarthropathy in the young, as in SL’s case. It is a rare condition, and mostly presents in the infancy with the characteristic cola coloured urine. However, diagnosis can be delayed, and patients can fall through the net. Patients can present in their late twenties with inflammatory low back pain, mimicking ankylosing spondylitis. Alkaptonuria has three cardinal features : homogentisic aciduria, ochronosis (visible pigmentation of eye, ear skin and teeth) and ochronotic spondyloarthropathy. Our patient had all of these classic features. HGA oxidises into benzoquinoneacetate, which turns into a melanin like polymer and gets deposited into tissues to cause the characteristic brown-black pigmentation. The commonly affected organs are large joints, heart and major blood vessels, skin, kidney and salivary glands. Patients may get recurrent pigment stones in kidney, prostate, gallbladder and salivary glands. Patients present with ochronotic arthropathy from the third decades onwards. Pigment deposited in the hyaline cartilage cause widespread joint destruction. Larger weight bearing joints are more commonly involved. It is also associated with early osteoporosis, as in Mr SL. Calcification of the spine along with osteoporosis increase the risk of fractures. Treatment of alkaptonuria remain mostly palliative. Physiotherapy,pain management remain the mainstay of treatment and arthroplasty may be required in the end Low protein diet and ascorbic acid are of unproven value. Nitisinone, which can reduce the HGA levels significantly, may be effective in some cases, however clinical trials failed to show efficacy. Further research is required to find an effective treatment for this rare condition.

**Key Learning Points:** Alkaptonuria is a rare autosomal recessive condition, classically presenting with cola coloured urine in infancy. It can mimic inflammatory low pain. It is associated with spondyloarthropathy, renal stones, pigmentation of skin and sclera. Blood and urine HGA levels can effectively diagnose this condition. Nitisinone has anecdotal evidence in the treatment of alkaptonuria, clinical efficacy in trials remain unproven

**Disclosure: S. Banerjee:** None. **H. Anver:** None. **J. Pinnell:** None. **P. Perkins:** None.

